# Effects and Mechanisms of Acupuncture on Diarrhea-Predominant Irritable Bowel Syndrome: A Systematic Review

**DOI:** 10.3389/fnins.2022.918701

**Published:** 2022-07-15

**Authors:** Gezhi Zhang, Tao Zhang, Zeng Cao, Zijing Tao, Tianhao Wan, Mengxi Yao, Xiaolan Su, Wei Wei

**Affiliations:** ^1^Department of Gastroenterology, Wangjing Hospital, China Academy of Chinese Medical Sciences, Beijing, China; ^2^Beijing Key Laboratory of Functional Gastrointestinal Disorders Diagnosis and Treatment of Traditional Chinese Medicine, Beijing, China

**Keywords:** brain-gut disorders, microbiota-gut-brain axis (MGB axis), acupuncture therapy, electroacupuncture therapy, transcutaneous electrical acustimulation, systematic review, diarrhea-predominant irritable bowel syndrome (IBS-D)

## Abstract

**Background:**

Irritable bowel syndrome (IBS) is a common disorder of gut-brain interaction with challenging treatment. According to evidence-based studies, acupuncture is likely to be a promising therapy and subservient adjunct for IBS. Mechanism study of acupuncture based on related clinical trials of high quality, nevertheless, is still vacant.

**Aim:**

This study aims to assess the results and qualities of current clinical evidence and conclude the relevant pathophysiological mechanisms and therapeutic effects of acupuncture on IBS with diarrhea (IBS-D).

**Methods:**

Literature from four databases, namely, PubMed, Cochrane Library, EMBASE, and Web of Science, was systematically searched to obtain eligible randomized controlled trials (RCTs), which contained mechanism research of acupuncture treatment in IBS-D patients. Two independent reviewers completed data extraction and quality evaluation using the RevMan 5.4.1 software.

**Results:**

Ten trials that covered 19 items related to mechanism research were included in this review. Acupuncture was reported to improve IBS-D symptoms and quality of life, with positive effects in regulating brain-gut peptides, cerebral activities, neuroendocrine functions, psychological state, and inflammatory GI and hypersensitive intestinal tracts.

**Conclusion:**

Acupuncture has potential influence on pathophysiology alterations such as regulating brain-gut peptides, altering cerebral connectivity and activity, promoting neuroendocrine functions and mental state, and mitigating inflammation as well as hypersensitivity of bowels in IBS-D patients, but further studies of high quality are still necessary.

**Systematic Review Registration:**

[https://www.crd.york.ac.uk/PROSPERO], identifier [CRD42022320331].

## Introduction

Irritable bowel syndrome (IBS) is a functional gastrointestinal (GI) disorder, or disorder of gut-brain interaction, affecting almost 9.2% of the general population (Oka et al., 2020). Without organic abnormalities, its clinical manifestations are characterized by abdominal pain or flatulence, accompanied by alteration in stool frequency or form (Mearin et al., 2016). IBS is divided into four subtypes according to Rome IV criteria (Drossman and Hasler, 2016), namely, IBS with constipation (IBS-C), IBS with diarrhea (IBS-D), IBS with a mixed pattern of constipation and diarrhea (IBS-M), and unclassified IBS, among which IBS-D is the most prevalent.

The pathogenesis of IBS-D is considered to be associated with genetic and environmental factors, but the particular mechanisms still remain unclear. IBS-D is regarded as a multifactorial disease, and stress was thought as the main cause of IBS-D (Labus et al., 2019); but with more and deeper investigations in recent years, the pathophysiology of IBS-D has been ascribed to multiple possibilities such as inflammatory GI, visceral hypersensitivity, genetic susceptibility, abnormal brain-gut interactions, and altered gut microbiota (Tang et al., 2021), rather than the simple response of the body to stress (Yaklai et al., 2021). Böhmelt et al. (2005) concluded that symptoms of IBS-D were related to the central dysfunction of viscerosomatic pathway led by the activation of immune-brain communication *via* vagal afferent fibers and the hypothalamic-pituitary-adrenal axis (HPA) (Böhmelt et al., 2005). To be more specific, the role of inflammatory cytokines imbalance cannot be ignored in IBS-D. Kumar et al. (2022) concluded that there seems to be a trend existing in the condition of increased levels of TNF-α and IL-6 and decreased level of IL-10. Besides, IL-10 is advocated as a potent cytokine target of anti-inflammatory therapy, especially in IBS-D. Visceral hypersensitivity induced by stress, reflected in the visceral pain threshold ordinarily, is also a reliable symbol (Nozu and Okumura, 2022). Present studies have demonstrated that genetic differences could be associated with IBS-D risk and symptoms (abdominal pain, sleep disturbance, and fatigue) (Zhao T. et al., 2022). Functional neuroimaging aids in the detection of brain alterations in IBS-D patients, and it is speculated that various functional disorders could have a shared pathophysiology (Nisticò et al., 2022). Referring to the microbiota-gut-brain axis, impaired intestinal mast cells led by the imbalance of intestinal flora excrete inflammatory mediators, and then, neurotransmitters are released, thus inducing aberrant intestinal motility and sensitivity (Chen et al., 2022). Thus, its complicacy on pathophysiological mechanisms resulted in restricted treatment methods, insufficient efficacy, and huge financial burdens.

As a common ailment with significant impact, IBS-D affects patients’ health as well as the quality of life (QOL), and the management of it mostly concentrates on symptomatic relief. Conventional remedies include pharmacological therapies (such as antibiotics, antagonists of serotonin 5-HT3 receptors, and antidepressants), dietary and lifestyle interventions, fecal microbiota transplantation (FMT), and other non-pharmacological approaches (Bonetto et al., 2021). However, most of these methods are always accompanied by some deficiencies or controversies. For instance, possible side effects of 5-HT3 antagonists are ischemic colitis and constipation (Adriani et al., 2018); another case in point is that the FMT was proved to have obvious clinical benefits in 8 single-arm trails but showed no superiority to placebo in 5 RCTs according to a meta-analysis (Myneedu et al., 2019). According to statistics from the United States, nearly two-thirds of IBS-D patients gave unsatisfactory comments on their current therapies (Zhang et al., 2022), the reason being the lack of efficacy or concomitant side effects. Despite the urgent demand for curative effect, the treatment of IBS-D is still challenging. Recently, a considerable part of patients tended to seek for complementary and interactive therapies, including acupuncture, moxibustion, traditional Chinese herbal medicines, and cognitive behavioral therapy. Although psychological interventions such as cognitive behavioral therapy or meditation have not shown obvious side effects, they are hard to be applied in long-term therapy for their specificity of manipulation.

Acupuncture, as one of the most popular complementary and alternative therapies, has been employed as an effective treatment for a great deal of common and tricky diseases. Originated in China, acupuncture has been successfully introduced into other countries. Data from the World Health Organization (WHO) showed that more than half of its member countries (103/194) had included acupuncture as a common treatment in their healthcare systems (Zhuang et al., 2013); 29 countries including Japan, Korea, the United States, and Canada had also approved legislation on acupuncture (Zhang et al., 2022). In the theoretical system of Traditional Chinese Medicine (TCM), acupuncture is defined as a therapeutic approach that regulates Qi (a TCM team that can be simply understood as a kind of energy) and blood through stimulating specific points (acupoints) in bodies. Modern relevant studies have found that its possible biological mechanisms are related to the microbiota-gut-brain (MGB) axis involving multiple factors such as neurotransmitters, immune regulation, oxidative stress, and intestinal flora (Zhang et al., 2022). Therefore, acupuncture has been applied to improve the imbalance of GMB in many diseases such as gastritis, colitis, obesity, and hypertension (Zheng et al., 2016; Wei et al., 2019). According to evidence-based studies (Huang et al., 2021; Zhao Y. et al., 2022), acupuncture is likely to be a promising therapy and subservient adjunct for IBS-D through modulating gut-brain axis and gut microbiome, but relevant evidence has not been systematically summarized until now. On the one hand, acupuncture was proved to exert more favorable effects than other interventions (pharmacological treatments) (Manheimer et al., 2012). On the other hand, acupuncture was compared with sham acupuncture but did not show a significant advantage in therapeutic efficacy (Lowe et al., 2017). Moreover, acupoints selection and parameters of acupuncture differ from each other in diverse studies, making it difficult to evaluate the authentic effects of acupuncture on IBS-D. Due to the low-quality evidence provided by systematic reviews and meta-analyses, the conclusion that acupuncture has exact beneficial effects for IBS-D still needs further confirmation.

On this account, to better understand the pathophysiological mechanisms and therapeutic effects of acupuncture on IBS-D in clinical studies, this study made efforts to summarize and assess concerning trials as comprehensively as possible. Based on diverse manipulations, the three most common types of acupuncture employed clinically, namely, manual acupuncture (MA), electroacupuncture (EA), and transcutaneous electrical acustimulation (TEA; Chen et al., 2018), are chosen in this article.

## Materials and Methods

### Protocol and Registration

The protocol of this systematic review was registered in the International Prospective Register of Systematic Reviews (PROSPERO),^1^ and the registration number is CRD42022320331.

### Selection Criteria

#### Study Designs

Randomized controlled trials (RCTs) published in peer-reviewed journals, with no restrictions on published date or language, were included.

#### Participants

Adults who were diagnosed with IBS-D under Rome I∼IV Criteria were included. There were no restrictions on gender or race.

#### Interventions

Trials that applied acupuncture therapy (mainly include MA, EA, and TEA; exclude acupoint moxibustion, acupoint embedding, and acupoint injection) in IBS-D patients were included. There were no limitations on the number of acupoints or the duration of treatment.

#### Comparators

Trials that compared sham acupuncture, moxibustion, medication, and no interventions with the experimental group were included (sham acupuncture and moxibustion should operate on the same acupoints as the experimental group).

#### Main Outcomes

Trials that conducted measurements containing mechanism research (serology such as inflammatory cytokines, visceral sensitivity, cerebral imaging, electroencephalogram, and questionnaires of mental status) on acupuncture were included.

### Search Strategy

A comprehensive search on relevant literature, the publication date of which was from inception to 20 March 2022, was accomplished in four databases, namely, PubMed, Cochrane Library, EMBASE, and Web of Science. Medical Subject Headings (MeSH) and entry terms of keywords were accessed from each database and then combined. The full search strategies of four databases were attached in the Supplementary Material.

### Study Selection and Data Extraction

Two researchers estimated the qualification of studies independently through browsing titles, abstracts, and full texts in proper order under the selection criteria above. Two researchers extracted data from included studies and checked the accuracy. Detailed information of extracted data is as follows: the first author, year of publication, study design, participant characteristics (including total number, gender, group allocation, and diagnostic standard), interventions (acupoints selection, duration, and frequency of treatment), main outcomes, and mechanism research. Any disagreement was resolved by discussion until consensus was reached or by consulting a third reviewer.

### Quality Assessment

Two researchers assessed the methodological quality and risk bias (random sequence generation, allocation concealment, blinding method, incomplete outcome data, selective reporting, and other bias) of all included studies using the RevMan 5.4.1 software (Cochrane Collaboration, Oxford, United Kingdom) based on *Cochrane Handbook for Systematic Reviews*. Any disagreement was resolved by discussion until consensus was reached or by consulting a third researcher.

## Results

### Study Selection

**FIGURE 1:**
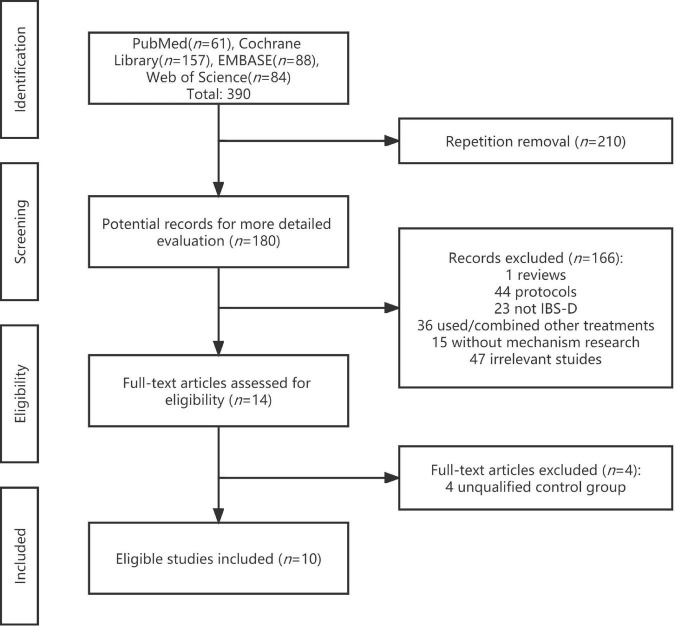
Process of study selection based on PRISMA.

The process of study selection based on PRISMA is shown in Figure 1. In total, 390 potential documents were retrieved from four databases, and of them, 180 documents remained after duplicate removal, which were to be examined through titles and abstracts soon afterward. Subsequently, 14 RCTs appeared to satisfy the included criteria and were carefully scanned with full texts. Two independent reviewers decided on the final 10 trials (Schneider et al., 2007; Chu et al., 2012; Wu et al., 2013; Zhan et al., 2014; Li, 2015; Zhao et al., 2015; Zhenzhong et al., 2015; Guo et al., 2021; Hu et al., 2021; Sun et al., 2021) to be included until consensus was reached.

### Study Characteristics

**FIGURE 2:**
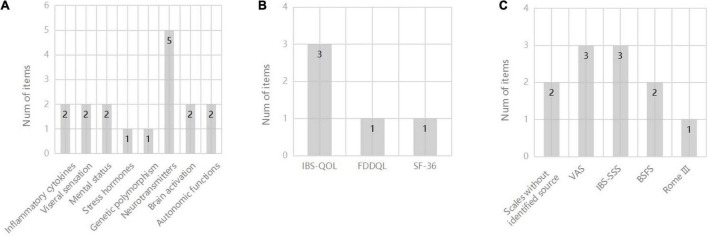
**(A)** Number of items for different scales of IBS-D symptoms; **(B)** Number of items for different scales of QOL; **(C)** Number of items for different mechanism research. BSFS, Bristol Stool Form Scale; FDDQL, functional digestive diseases quality of life; IBS-QOL, IBS quality of life; IBS-SSS, IBS severity scoring system; SF-36, 36-Item Short Form Survey; VAS, visual analog scale.

Characteristics of ten included studies are shown in Table 1. Publication date of the ultimate 10 studies in this review ranged from 2007 to 2022, and a total of 665 participants were involved while the exact number of each study varied from 30 to 231. The percentage of females in these participants was 51.4–82.4%, unknown in two trials (Zhao et al., 2015; Zhenzhong et al., 2015). The intervention methods in experimental groups were all acupuncture, including MA, EA, and TEA. Three of them (Schneider et al., 2007; Chu et al., 2012; Hu et al., 2021) took sham acupuncture as contrast, compared to four (Wu et al., 2013; Zhan et al., 2014; Li, 2015; Guo et al., 2021) using medication treatment and two (Zhao et al., 2015; Zhenzhong et al., 2015) with moxibustion. Nine studies (Schneider et al., 2007; Wu et al., 2013; Zhan et al., 2014; Li, 2015; Zhao et al., 2015; Zhenzhong et al., 2015; Guo et al., 2021; Hu et al., 2021; Sun et al., 2021) adopted certain gastrointestinal symptom scales to reflect the acupuncture efficacy in IBS-D (see Figure 2A), and quality of life scales (QOL) were put to use in four trials (Schneider et al., 2007; Guo et al., 2021; Hu et al., 2021; Sun et al., 2021) (see Figure 2B). As for mechanism research, 19 items in aggregate from manifold categories (inflammatory cytokines, genetic polymorphism, mental status, visceral sensation, stress hormones, neurotransmitters and their receptors, autonomic functions and brain activation) were investigated in studies (see Figure 2C). Detailed information of study characteristics was shown in Table 2.

**TABLE 1:** Characteristics of included studies of acupuncture vs. sham acupuncture, medication, and moxibustion in treating Diarrhea-Predominant Irritable Bowel Syndrome.

References	Design	Participants	Interventions	Main outcomes	Mechanism research
Hu et al., 2021	Parallel	*n*: 37 (F 19); Age (mean): TEA (44.4 ± 13.4), CONT (45.4 ± 11.7); Groups (*n*): TEA (21), CONT (16); Diagnosis: Rome IV	EXP: TEA (LI4, ST36); CONT: Sham TEA (LI4, ST36, no current delivered); Duration and frequency: EXP&CONT: 30 min, twice per d, for 1 month	Decreased VAS scores in TEA and greater than SA (*P* < 0.05); improved IBS-QOL scores in TEA not in SA; decreased IBS-SSS scores in both TEA and SA	Inflammatory cytokines (Serum; IL-10 and IL-6); Neurotransmitters (Serum, NE); GI hormones (Serum, PP)
Guo et al., 2021	Parallel	*n*: 231 (F 103); Age (mean): MA (46 ± 12), CONT (44 ± 13); Groups (*n*): MA (154), CONT (77); Diagnosis: Rome III	EXP: MA (GV20, GV29, ST25, ST36, ST37, SP6, LR3); CONT: Oral pinaverium bromide tablets; Duration and frequency: EXP: 30 min, once every other d, for 6 wk; CONT: 50 mg, tid, for 6 wk	Decreased IBS-SSS scores in MA and greater than CONT (*P* < 0.01); improved IBS-QOL in MA and greater than CONT (*P* < 0.01)	Genetic polymorphism (5-HTTLPR)
Sun et al., 2021	Parallel	*n*: 73 (F 39); Age (mean): MA + EA (39 ± 10), EA (41 ± 10); Groups (*n*): MA + EA (36), EA (37); Diagnosis: Rome IV	EXP: MA (GV20, GV24, GB13); CONT: MA + EA (CV4, CV12, ST25, BL25, ST36, ST37, LI4, LR3); Duration and frequency: EXP&CONT: 30 min, once per d, 6 times per wk, for 4 consecutive wk	Decreased IBS-SSS scores in MA + EA and greater than EA (*P* < 0.05); improved IBS-QOL in MA + EA and greater than EA (*P* < 0.05)	Mental status (HAMD)
Li, 2015	Parallel	n: 60 (F 33); Age (mean): MA (32), CONT (34); Groups (*n*): MA (30), CONT (30); Diagnosis: Rome III	EXP: MA (CV6, CV12, ST25, ST36, ST37, ST39, SP6, SP7, SP9); CONT: Oral pinaverium bromide tablets; Duration and frequency: EXP: 25 min, once per d, 5 times per wk, for 4 wk; CONT: 50mg, tid, for 4 wk	Decreased symptom scores in MA and greater than CONT (*P* < 0.05) (symptom scores without identified source)	Neurotransmitters (Serum; VIP and 5-HT)
Zhenzhong et al., 2015	Parallel	*n*: 41 (F NA); Age (mean): EA (39 ± 5), Mox (39 ± 8); Groups (*n*): EA (19), Mox (22); Diagnosis: Rome III	EXP: EA (ST36, ST37); CONT: Moxibustion (ST36, ST37); Duration and frequency: EXP&CONT: 30 min, once per d, 6 times per wk, for 4 consecutive wk	Decreased VAS scores in both EA and Mox; decreased BSFS scores in Mox and greater than EA (*P* < 0.001)	Neurotransmitters (Colon tissue; SP and VIP)
Zhao et al., 2015	Parallel	*n*: 62 (F NA); Age (mean): EA (42.75 ± 10.22), Mox (39.53 ± 8.91); Groups (*n*): EA (32), Mox (30); Diagnosis: Rome III	EXP: EA (ST25, ST37); CONT: Moxibustion (ST25, ST37); Duration and frequency: EXP&CONT: 30 min, once per d, 6 times per wk, for 4 consecutive wk	Decreased VAS scores in both EA and Mox; decreased BSFS scores in Mox not in EA; decreased HAMD and HAMA scores in both EA and Mox (*P* < 0.05 or *P* < 0.01)	Mental status (HAMD and HAMA); Neurotransmitters (Sigmoid tissue; 5-HT, 5-HT3R and 5-HT4R); Brain activation (fMRI)
Zhan et al., 2014	Parallel	*n*: 57 (F 36); Age (mean): MA (42 ± 14), CONT (37 ± 13); Groups (*n*): MA (29), CONT (28); Diagnosis: Rome III	EXP: MA (GV20, GV29, LR3, ST25, ST36, ST37, SP6); CONT: Oral live combined bifidobacterium and lactobacillus tablets/pinaverium bromide tablets; Duration and frequency: EXP: 30 min, once per d, 5 times per wk, for 4 wk; CONT: 2 g, tid/50 mg, tid, for 4 wk	Decreased Rome III IBS symptom scores in MA and greater than CONT (*P* < 0.01); significantly higher PR in MA than CONT (*P* < 0.05)	Neurotransmitters (Serum; 5-HT)
Wu et al., 2013	Parallel	*n*: 40 (F 22); Age (mean): MA (41 ± 13), CONT (39 ± 13); Groups (*n*): MA (21), CONT (19); Diagnosis: Rome III	EXP: MA (GV20, GV29, LR3, ST25, ST36, ST37, SP6); CONT: Oral live combined bifidobacterium and lactobacillus tablets/pinaverium bromide tablets; Duration and frequency: EXP: 30 min, once per d, 5 times per wk, for 4 wk; CONT: 2 g, bid/50 mg, tid, for 4 wk	Decreased symptom scores in MA and greater than CONT (*P* < 0.05) (symptom scores without identified source); significantly higher PR in MA than CONT (*P* < 0.05)	Inflammatory cytokines (Serum; IFN-γ, IL-2, IL-4, IL-10 and IFN-γ/IL-4)
Chu et al., 2012	Parallel	*n*: 30 (F 15); Age (mean): EA (42.3 ± 12.2), SA (44.2 ± 14.5); Groups (*n*): MA (15), CONT (15); Diagnosis: Rome III	EXP: EA (ST36, ST37, SP6); CONT: Sham EA (ST36, ST37, SP6; touch but not penetrate into acupoints); Duration: EXP&CONT: 15 and 30 min, once during fMRI	−	Brain activation (fMRI)
Schneider et al., 2007	Parallel	*n*: 34 (F 28); Age (mean): MA (46.23 ± 15.00), SA (41.80 ± 14.51); Groups (*n*): MA (19), CONT (15); Diagnosis: Rome II	EXP: MA (LR3, ST21, ST25, ST36, SP6, HT7, GV20, RN12); CONT: Sham MA (LR3, ST21, ST25, ST36, SP6, HT7, GV20, RN12; 2 cm adjacent to acupoints); Duration and frequency: EXP&CONT: twice a wk, for 5 wk	Decreased FDDQL global scores and SF-36 pain scales in both MA and SA	Stress hormones (salivary cortisol); Autonomic functions (ECG)

*BSFS, Bristol Stool Form Scale; CONT, control group; EA, electroacupuncture; ECG, electrocardiogram; EXP, experimental group; F, female; FDDQL, functional digestive diseases quality of life; fMRI, functional magnetic resonance imaging; HAMA, Hamilton Anxiety Rating Scale; HAMD, Hamilton Depression Rating Scale; IBS-QOL, IBS quality of life; IBS-SSS, IBS severity scoring system; IFN-γ, interferon γ; IL, interleukin; MA, manual acupuncture; Mox, moxibustion; NE, norepinephrine; PP, pancreatic polypeptide; PR, proportion of responders; SA, sham acupuncture/TEA; SF-36, 36-Item Short Form Survey; SP, substance P; TEA, transcutaneous electrical acustimulation; VAS, visual analog scale; VIP, vasoactive intestinal peptide; 5-HT, 5-hydroxytryptamine; 5-HT3R, serotonin receptor 3; 5-HT4R, serotonin receptor 4; 5-HTTLPR, serotonin transporter.*

**TABLE 2:** Mechanism research of included studies.

Reference	Detecting items	Research methods and techniques	Main results
** Brain-gut peptides (neurotransmitters and GI hormones)**			
Hu et al., 2021	Serum NE and PP levels	Corresponding commercial kits	No significant alteration in NE and PP levels with TEA nor with sham TEA
Li, 2015	Serum VIP and 5-HT levels	ELISA	Decreased serum VIP and 5-HT levels in MA and greater than CONT (P < 0.05)
Zhenzhong et al., 2015	Colon tissue SP and VIP levels	Immunohistochemical staining	Decreased SP and VIP expression in both EA and Mox
Zhao et al., 2015	Sigmoid tissue 5-HT, 5-HT3R and 5-HT4R levels	Immunohistochemical staining	Decreased 5-HT3R and 5-HT4R expression in both EA and Mox; decreased 5-HT expression in Mox and greater than EA (*P* < 0.05)
Zhan et al., 2014	Serum 5-HT levels	ELISA	Decreased serum 5-HT levels in both MA and CONT
** Cerebral activities**			
Zhao et al., 2015	Brain activation	fMRI	Decreased voxel values of PFC in EA
Chu et al., 2012	Brain activation	fMRI	Significantly higher activation at right insula, pulvinar and medial nucleus of the thalamus was observed in EA compared to SA
** Neuroendocrine functions**			
Hu et al., 2021	Serum NE and PP levels	Corresponding commercial kits	No significant alteration in NE and PP levels with TEA nor with sham TEA
Schneider et al., 2007	Salivary cortisol levels Heart rate and blood pressure	Radioimmune assay ECG	Decreased cortisol concentrations in MA not in SA Parasympathetically decreased heart rate response in MA not in SA during orthostasis
*** Mental status***			
Sun et al., 2021	Depression	HAMD	Decreased HAMD scores in MA + EA and greater than EA (*P* < 0.05)
Zhao et al., 2015	Depression and anxiety	HAMD and HAMA	Decreased HAMD and HAMA scores in both EA and Mox (*P* < 0.05 or *P* < 0.01)
** Inflammation and hypersensitivity of the bowels**			
Hu et al., 2021	Serum IL-10 and IL-6 levels	Corresponding commercial kits	No significant alteration in IL-10 and IL-6 levels with TEA nor with sham TEA
Wu et al., 2013	Serum IFN-γ, IL-2, IL-4, IL-10 and IFN-γ/IL-4	Not mentioned	Improved serum IL-4 and IL-10 in MA and greater than CONT (P < 0.05); decreased serum IFN-γ/IL-4 in MA and greater than CONT (*P* < 0.01)
Zhao et al., 2015	Rectal sensory thresholds	VAS	Significant increases in the urgent defecation perception thresholds and maximum pain perception thresholds were observed in both EA and Mox groups after treatment (*P* < 0.05 or *P* < 0.01)
Chu et al., 2012	Rectal sensation	Likert scale	A significant correlation (*P* < 0.005) between subjective rectal pain rating and brain activation was observed in hypothalamus, bilateral thalami and bilateral insula
**Genetic polymorphism**			
Guo et al., 2021	5-HTTLPR	Genetic polymorphism	SS genotypes take over a higher proportion in IBS-D patients; curative effect of acupuncture was better in LS and SS genotypes than LL and the same genotype in CONT

*CONT, control group; EA, electroacupuncture; ECG, electrocardiogram; ELISA, enzyme-linked immunosorbent assay; fMRI, functional magnetic resonance imaging; HAMA, Hamilton Anxiety Rating Scale; HAMD, Hamilton Depression Rating Scale; IFN-γ, interferon γ; IL, interleukin; MA, manual acupuncture; LS, SS, and LL, three genotypes in 5-HTTLPR; Mox, moxibustion; NE, norepinephrine; PFC, prefrontal cortex (the senior center of feeling pain); PP, pancreatic polypeptide; SA, sham acupuncture/TEA; SP, substance P; TEA, transcutaneous electrical acustimulation; VAS, visual analog scale; VIP, vasoactive intestinal peptide; 5-HT, 5-hydroxytryptamine; 5-HT3R, serotonin receptor 3; 5-HT4R, serotonin receptor 4; 5-HTTLPR, serotonin transporter.*

### Clinical Effects

#### Gastrointestinal Symptoms and Quality of Life

Nine studies indicated decreased GI symptom scores after the acupuncture treatment based on multiple methods and symptom scales (Figure 2A), making it difficult to evaluate these results in a synthetic way. Four of these trials reported improvements in QOL under acupuncture treatment, and the IBS-QOL was the most selected scale to measure GI symptoms in IBS-D among the studies (Figure 2B).

### Pathophysiology

#### Inflammatory Cytokines

Five inflammatory cytokines were tested in two trials (Wu et al., 2013; Hu et al., 2021), including interleukin 10 (IL-10), IL-6, IL-4, IL-2, and interferon-γ (INF-γ). All the items were assessed from the blood samples using corresponding commercial kits. In the study by Hu et al. (2021), IL-10 and IL-6 were not significantly changed after TEA treatment. The other trial (Wu et al., 2013) took INF-γ, IL-2, IL-4, and IL-10 as targets and demonstrated that IL-4 and IL-10 levels were obviously increased, while no significant alteration was observed in INF-γ and IL-2 levels.

#### Visceral Sensation

Two studies conducted rectum distension and recorded the maximum tolerable thresholds pressure during the trial (Chu et al., 2012; Zhao et al., 2015). Zhao et al. (2015) put a plastic balloon in patients’ rectum and progressively injected gas into it, while Chu et al. (2012) accomplished that with a computer-driven barostat. Their results were quite different. The former proved that significant increase in maximum pain perception thresholds was observed after EA treatment, but the latter indicated no obvious change.

#### Mental Status

Depression and anxiety were two main objects to appraise mental status. Two studies evaluated depression and anxiety state with *Hamilton Depression Rating Scale* (HAMD) and/or *Hamilton Anxiety Rating Scale* (HAMA), reporting improvement in psychological condition after MA and EA.

#### Stress Hormones

Measurement of salivary cortisol was processed by radioimmune assay in one study (Schneider et al., 2007). After acupuncture treatment, the cortisol concentrations showed significant reduction at all assessment points.

#### Genetic Polymorphism

The serotonin transporter polymorphism (5-HTTLPR) genotypes were determined by means of DNA amplification with polymerase chain reaction (PCR) in one study (Guo et al., 2021). The results indicated that there could be a correlation between clinical efficacy of acupuncture and 5-HTTLPR polymorphism, and the efficacy is more obvious in patients with LS and SS genotypes.

### Mechanisms

#### Neurotransmitters and Their Receptors

Six neurotransmitters were tested in five of the ten trials (Zhan et al., 2014; Li, 2015; Zhao et al., 2015; Zhenzhong et al., 2015; Hu et al., 2021), including norepinephrine (NE), vasoactive intestinal peptide (VIP), substance P (SP), 5-hydroxytryptamine (5-HT), and serotonin receptor 3 and receptor 4 (5-HT3R/5-HT4R). Enzyme-linked immunosorbent assay (ELISA) and immunohistochemical (IHC) staining were applied as testing methods in studies, from which MA/EA was reported to decrease VIP, SP, 5-HT, and its receptors levels, but NE levels alteration was not significant after TEA.

#### Brain Activation

By means of functional magnetic resonance imaging (fMRI), one study observed decreased activated voxel values in the functional brain areas of the prefrontal cortex (PFC, the senior center of feeling pain) under stimulation with colorectal distension (CRD) after EA treatment (Zhao et al., 2015). In addition, another study, treated with EA as well, reported a significantly higher activation in the right insula, pulvinar, and medial nucleus of the thalamus under fMRI (Chu et al., 2012).

#### Autonomic Functions

The NE and pancreatic polypeptide (PP) were assessed in one study using a reagent test kit (Hu et al., 2021), to reflect the sympathetic activity and vagal activity. After TEA treatment, however, NE and PP levels did not vary significantly. Besides, one more trial showed an acupuncture-induced decrease in heart rate response through electrocardiogram (ECG) during orthostatic stress (Schneider et al., 2007), which indicated an increase in parasympathetic tone.

### Methodological Quality

**FIGURE 3:**
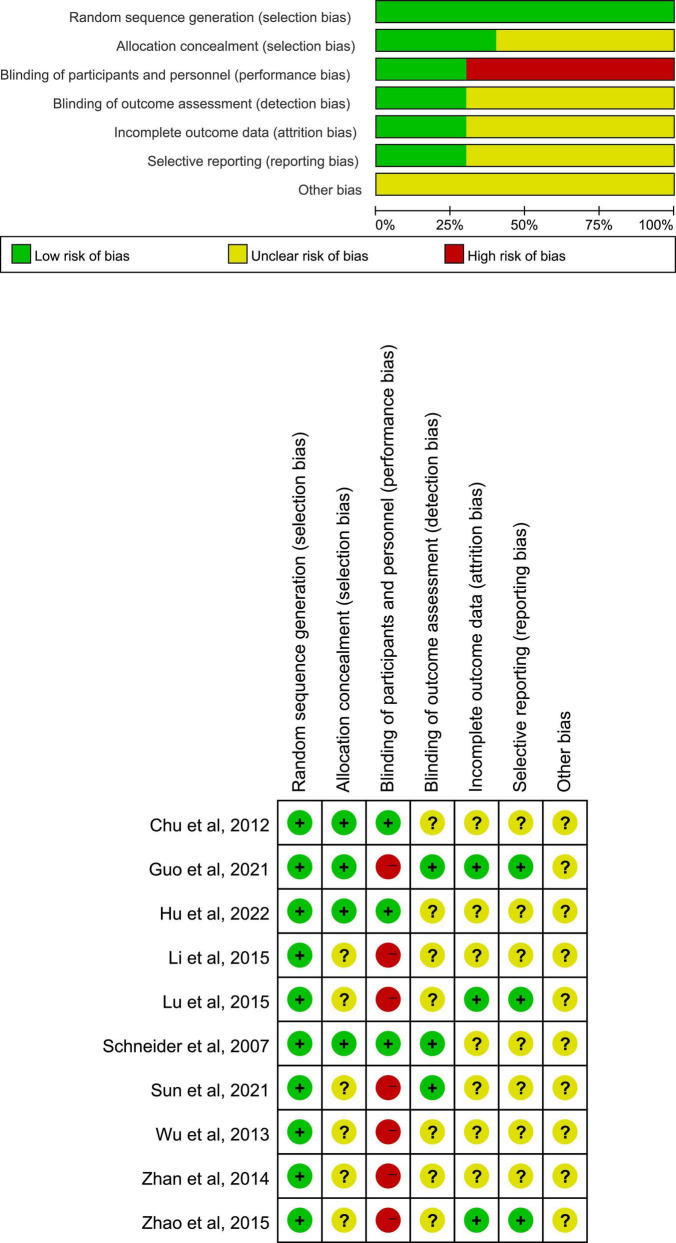
Risk of bias graph and summary of included studies.

The risk of bias graph and summary of assessment on methodological quality about included studies were displayed in Figure 3. All studies showed a low risk in selection bias (random sequence generation). The risk of selection bias (allocation concealment), detection bias (blinding of outcome assessment), attrition bias (incomplete outcome data), and reporting bias (selective reporting) was unclear in most trials due to the lack of detailed illustrations or experiment protocols previously. A proportion of studies manifested a high risk in performance bias (blinding of participants and personnel), owing to the non-acupuncture control groups such as oral medicine and moxibustion.

## Discussion

In view of the sophisticated and multifactorial pathophysiology, general symptomatic treatment like pharmacotherapy is incapable to appease the momentous requirement for care in IBS-D patients (De Ponti, 2013). It has been calculated that 30–50% of patients with IBS-D take complementary and alternative medicine (CAM) therapies to relieve their symptoms (Nguyen, 2018), and clinical evidence supporting the utilization of acupuncture is demonstrating that CAM interventions like this present improvement in IBS-D overall symptoms and QOL indeed (Grundmann and Yoon, 2014). Acupuncture has been widely used in the treatment of GI dysfunctions such as IBS-D, inflammatory bowel disease, and functional dyspepsia. As the most classical method, MA is commonly applied in various modes, which might exert quite different impacts on one disease (Guo Y. et al., 2020). Compared with the uncertain factitiousness of MA, EA seems to be a more reliable approach due to the additional neuromodulation conducted by machines. It is approved that electrical stimulation from EA improves nausea and vomiting, encelialgia, delayed gastric emptying, and myoelectric activity (Sarosiek et al., 2017). TEA replaces the needles of EA with self-adhesive electrodes laid on acupoints (Qu et al., 2017) and becomes a more accepted treatment for its convenience and economy (Chen et al., 2018). Acupoints have been emphasized as crucial elements for the effects of acupuncture, and by stimulating the specific acupoints, the required efficacy can be achieved. Several studies have reviewed and concluded the most commonly used acupoints for IBS-D, including ST25, ST36, CV12, LR3, SP6, GV20, etc. (Zhu et al., 2018; Yan et al., 2019; Su et al., 2021; Zhang et al., 2022). Acupuncture has been approved to realize the symptom relief and the quality-of-life improvement in mild and moderate IBS-D according to a Delphi expert consensus (Su et al., 2021). Mechanism study of acupuncture based on IBS-related clinical trials of high quality, nevertheless, is still insufficient. The primary aim of this review is to summarize and reveal the underlying mechanisms of acupuncture treating IBS-D based on the studies published at present.

We combined RCTs with mechanism studies of acupuncture in remedying IBS-D patients in this systematic review and concluded the extant indications that were likely to provide conceivable explanations for the curative effects of acupuncture by evidence-based evaluation. In this review, we found that a majority of RCTs laid their emphasis on brain-gut peptides, cerebral activities, neuroendocrine functions, psychological state, and inflammatory GI and hypersensitive intestinal tracts to clarify potential mechanisms of acupuncture. All ten studies pointed out that acupuncture therapies, such as MA, EA, and TEA, were of certain benefit in relieving IBS-D symptoms, and four studies reported improvement in the QOL of IBS, which was in accordance with a previous investigation (Guo J. et al., 2020) in the rough.

The IBS-D is defined as a disorder of gut-brain interaction since Rome IV came out (Drossman et al., 2018). In pace with further studies, it is also labeled as a disturbance of MGB axis (Hillestad et al., 2022), which consists of gut microbiota, intestinal epithelial barrier, neurotransmitters, enteric nervous system (ENS), central nervous system (CNS), autonomic nervous system, and hypothalamic-pituitary-adrenal (HPA) axis, participating in the bidirectional communication between gut and brain (Gros et al., 2021). At present, the available proof has attested the important role of brain-gut peptides (including neurotransmitters and GI hormones) in regulating the MGB axis on IBS-D pathophysiology, in consideration that many physiological manifestations such as GI motility abnormalities, visceral paresthesias, central disorders, and psychosocial factors (anxiety and depression) are associated with them (Mayer et al., 2019; Chen et al., 2022). Serotonin (also called 5-hydroxytriptamin, 5-HT) that serves as a critical neurotransmitter in the body and as a paracrine messenger in GI (Mittal et al., 2017) is reported to control neurological functions and modulate GI secretion, peristalsis, and absorption plus visceral hyperalgesia (Spohn and Mawe, 2017). Serotonin receptors are widely believed to play a part in anxiety and depression performances, among which the inhibition of 5-HT3R and the activation of 5-HT4R could realize anti-depressant effects (Żmudzka et al., 2018). In addition, 5-HT3R intervenes in the gut-brain communication, modulating gut motility and visceral pain signaling (Breit et al., 2018); both 5-HT3R and 5-HT4R mediate gastric accommodation (Gros et al., 2021). Imbalance in norepinephrine (NE) along with serotonin levels influences the comorbidity between emotional distress to a great extent (Chávez-Castillo et al., 2019), but relevant studies on investigating the function of NE are limited (Barandouzi et al., 2022). Intestinal NE can enhance the growth of *Escherichia coli* (*E. coli*) and promote intestinal motility to form biofilm and virulence of *E. coli* (Jubelin et al., 2018). As one of the main mediators of stress, which could be a risk factor for IBS-D, a high NE level is correlated with anxiety disorders (Gros et al., 2021). In terms of alteration in GI motility, vasoactive intestinal peptide (VIP) and substance P (SP) are bound to be mentioned, which also involve pathological reactions such as abdominal pain or discomfort, abnormal defecation, and visceral hypersensitivity (Bednarska et al., 2017). As a GI hormone, pancreatic polypeptide (PP) is deemed as a marker to evaluate vagal efferent activity (Simonian et al., 2005), and studies showed that the PP level dropped in the TEA group (Song et al., 2018). In this review, four studies demonstrated a reduction in 5-HT, 5-HT3R, 5-HT4R, VIP, and SP levels after MA/EA treatment (Zhan et al., 2014; Li, 2015; Zhao et al., 2015; Zhenzhong et al., 2015). Hu et al. (2021) found that NE levels were not changed significantly with TEA; however, the potential autonomic mechanism of it should not be ignored because factors like differences in manipulation (selection of stimulation parameters and acupoints), experimental conditions, or patient population could probably affect autonomic functions. Furthermore, TEA-induced reduction of NE showed a borderline negative correlation with improvement in abdominal pain, suggesting that over-dynamic sympathetic nerve might exert a limited function on abdominal pain.

To help understand the pathophysiological mechanisms of IBS-D, functional magnetic resonance imaging (fMRI) has been applied in a large proportion of studies, which put their emphasis on structural and functional brain connectivity (Kano et al., 2020). Some studies based on psychological abnormalities in IBS-D patients indicated a correlation between GI symptoms, severity of mentation and unusual activation of certain cerebral regions (Drossman et al., 2003) (enhanced or decreased activation in the ACC, IC and PFC (Silverman et al., 1997)). One study in this review showed decreased activation of PFC after EA treatment (Zhao et al., 2015); another study formulated that acupuncture might exert potential influence on the modulation of 5-HT pathway in insula and mood *via* ascending pathway in the higher cortical center (pulvinar and medical nucleus of the thalamus) to process pain in IBS-D (Chu et al., 2012). As for autonomic functions, NE and PP levels, which were used to reflect sympathetic activity and vagal activity, respectively, in a study (Hu et al., 2021), did not change significantly with TEA; another study showed decreased heart rate response and increased parasympathetic tone through ECG with MA. In addition, there was a significant relation between the decrease in heart rate response and increase in pain-related QOL (SF-36) in MA (Hu et al., 2021). An early trial displayed an augmented parasympathetic tone accompanied by the palpable abatement of salivary cortisol and alleviation of pain in response to acupuncture treatment, which coincides with the overactive axis in IBS-D patients (Posserud et al., 2004).

Anxiety and depression are thought to exist in 30–50% of patients with chronic GI symptoms (Lee et al., 2017) and are associated with the brain-gut axis, suggesting that more emphasis should be attached to the inspection and management of neuropsychiatric symptoms (Dao et al., 2021). Empirical evidence declares that such psychosomatic symptoms lead to a double acceleration in the morbidity of GI symptoms as well (Takajo et al., 2019). Two studies applied Hamilton Depression Rating Scale (HAMD) and/or Hamilton Anxiety Rating Scale (HAMA) to estimate the mental status of IBS-D patients and reported a pronounced decrease in scores after MA and/or EA (Zhao et al., 2015; Sun et al., 2021).

Two studies tried to validate the anti-inflammatory effect of TEA and MA through detecting cytokines. One trial assessed the serum levels of IL-6 and IL-10, which were pro-inflammatory and anti-inflammatory cytokines, respectively, and showed that TEA treatment did not significantly change neither IL-6 nor IL-10 levels in comparison with sham TEA (Hu et al., 2021). The other trial took peripheral Th1 cytokine INF-γ and IL-2, Th2 cytokines IL-4 and IL-10 as targets and demonstrated that anti-inflammatory cytokines (IL-4, IL-10) levels were obviously elevated under acupuncture treatment, while no significant alteration was observed in pro-inflammatory cytokines (INF-γ and IL-2), verifying the upregulation effect of acupuncture on Th2 cytokines level and the recovery on imbalanced Th1/Th2 (Wu et al., 2013). It has been speculated by a previous researcher (Zhang et al., 2018) that TEA might activate peripheral nerves and deliver a signal to the center where the cerebrum processes signals and exports a stronger vagal efferent semaphore, and subsequently, the gut releases acetylcholine to make the secretion of inflammatory cytokines back to a balance. Abdominal pain and distension are typical characteristics of visceral hypersensitivity; another two studies measured the sensibility of bowels *via* proctectasia. One of them performed enhanced urgent defecation perception thresholds and maximum pain perception thresholds with EA (Zhao et al., 2015); the other study showed no distinct difference in pain tolerance, the possible reason of which was attributed to a single session of EA by the author (Chu et al., 2012). In the light of numerous findings, IBS-D has a bearing on increased irritability of esthesioneure in the gut. EA regulates intestinal motility, intestinal microflora (Song et al., 2020), visceral receptor sensitivity, and brain-gut axis (Chen et al., 2019), so as to mitigate the hypersensitive condition in irritable bowels (Hu et al., 2021).

The differential transcriptional activity caused by polymorphism in the promoter region of the gene coding 5-HTT (5-HTTLPR) was speculated to affect complicated symptoms and diseases including IBS-D and affective disorders (Goldman et al., 2010). Previous scholars have proved that 5-HTTLPR polymorphism affects the activity of serotonin reuptake transporter (SERT) and closely relates to the pathogenesis as well as symptom burdens of IBS-D (Jia et al., 2019; Zhao T. et al., 2022). One study in this review determined the 5-HTTLPR genotypes of patients before acupuncture therapy, and they were classified into three types, namely, LL, LS, and SS (L stands for long and S stands for short). The results indicated the correlation between the curative effect of MA and 5-HTTLPR polymorphism, and it was more evident in LS and SS crowds.

### Limitations and Expectations

Several limitations in this review are as follows: (1) Heterogenicities among the included trials. In view of the diverse designs (acupoints protocols), outcomes, evaluation methods, and mechanisms in these studies, it is difficult to merge the cognate data for further meta-analysis. (2) Inadequate high-quality methodological studies. Critical clues such as allocation concealment and blinding of outcome assessment remain unavailable, which is possibly leading to untrustworthy results and conclusions, especially under the undefined placebo effects of acupuncture. Additionally, most included trials were performed in one country (Zhuang et al., 2013; Zheng et al., 2016; Adriani et al., 2018; Myneedu et al., 2019; Wei et al., 2019; Huang et al., 2021; Zhang et al., 2022; Zhao Y. et al., 2022), accompanied by equivocal reporting bias. (3) Ignorance of the impacts of other diseases. Most included studies focus on intestinal tract symptoms to find out the mechanism of acupuncture, thus neglecting the other factors. (4) Insufficient types of outcome evaluation methods. For instance, there is no study among these trials that utilize intestinal flora or food sensitivities as endpoints to assess the effects of acupuncture. To ameliorate these deficiencies, future studies should make more efforts in experimental design and multi-area investigation. Mechanism studies about acupuncture should lay more emphasis on the MGB axis so as to keep abreast of the latest cognition and consensus on pathophysiology in IBS-D.

## Conclusion

In conclusion, this systematic review demonstrated the qualities and results of relevant RCTs, indicating that acupuncture therapies (MA, EA, and TEA) might improve the IBS-D symptoms through regulating brain-gut peptides, altering cerebral connectivity and activity, promoting neuroendocrine functions and mental state, and mitigating inflammation as well as hypersensitivity of bowels. In consideration that most current studies only fix their attention on one or a few focal points, the chain of evidence about mechanisms of acupuncture on IBS-D patients seems to be quite scattered. This review tried to summarize and integrate existing evidence through available RCTs, hoping to provide acupuncturists and researchers with some reliable information about the mechanisms of acupuncture on IBS-D.

## Data Availability Statement

The original contributions presented in this study are included in the article/Supplementary Material, further inquiries can be directed to the corresponding authors.

## Author Contributions

XS designed the review protocol. GZ conducted the literature research and drafted the manuscript. GZ and TZ contributed to the data extraction. ZC and ZT contributed to the quality assessment. TW and MY contributed to the methodological guidance. XS and WW contributed to the critical revision of the manuscript. All authors contributed to the article and approved the submitted version.

## Conflict of Interest

The authors declare that the research was conducted in the absence of any commercial or financial relationships that could be construed as a potential conflict of interest. The reviewer YW declared a shared affiliation with the authors at the time of review.

## Publisher’s Note

All claims expressed in this article are solely those of the authors and do not necessarily represent those of their affiliated organizations, or those of the publisher, the editors and the reviewers. Any product that may be evaluated in this article, or claim that may be made by its manufacturer, is not guaranteed or endorsed by the publisher.

## Footnotes

1
https://www.crd.york.ac.uk/PROSPERO
