# Supplementary material for: Effects and Mechanisms of Acupuncture on Diarrhea-Predominant Irritable Bowel Syndrome: A Systematic Review
Source: Front Neurosci. 2022 Jul 15;16:918701. doi: 10.3389/fnins.2022.918701 (PMC9334728; doi:10.3389/fnins.2022.918701)
Supplement: Supplementary file 1 [file Data_Sheet_1.pdf]

Full Search strategies

PubMed

#1 ("Acupuncture"[Mesh]) OR (Acupuncture[Title/Abstract])

#2 ("Electroacupuncture"[Mesh]) OR (Electroacupuncture[Title/Abstract])

#3 ("Transcutaneous Electric Nerve Stimulation"[Mesh]) OR (((((Transcutaneous Electric Nerve Stimulation[Title/Abstract]) OR (Transcutaneous Electric Stimulation[Title/Abstract])) OR (Transdermal Electrostimulation[Title/Abstract])) OR (Percutaneous Electric Nerve Stimulation[Title/Abstract])) OR (Percutaneous Electrical Neuromodulation[Title/Abstract]))

#4 ("Irritable Bowel Syndrome"[Mesh]) OR ((Irritable Bowel Syndrome[Title/Abstract]) OR (Irritable Colon[Title/Abstract]))

#5 #1 OR #2 OR #3

#6 #4 AND #5

Cochrane Library

#1 MeSH descriptor: [Acupuncture] explode all trees

#2 (Acupuncture):ti,ab,kw

#3 MeSH descriptor: [Electroacupuncture] explode all trees

#4 (Electroacupuncture):ti,ab,kw

#5 MeSH descriptor: [Transcutaneous Electric Nerve Stimulation] explode all trees

#6 (Transcutaneous Electric Nerve Stimulation):ti,ab,kw OR (Transcutaneous Electric Stimulation):ti,ab,kw OR (Transdermal Electrostimulation):ti,ab,kw OR (Percutaneous Electric Nerve Stimulation):ti,ab,kw OR (Percutaneous Electrical Neuromodulation):ti,ab,kw

#7 MeSH descriptor: [Irritable Bowel Syndrome] explode all trees

#8 (Irritable Bowel Syndrome):ti,ab,kw OR (Irritable Colon):ti,ab,kw

#9 (#1 OR #2) OR (#3 OR #4) OR (#5 OR #6)

#10 #7 OR #8

#11 #9 AND #10

Embase

#1 'acupuncture'/exp OR acupuncture:ti,ab,kw

#2 'electroacupuncture'/exp OR electroacupuncture:ti,ab,kw

#3 'transcutaneous electrical nerve stimulation'/exp OR 'transcutaneous electrical nerve stimulation':ti,ab,kw OR 'transcutaneous electrical stimulation':ti,ab,kw OR 'transcutaneous electrostimulation':ti,ab,kw OR 'percutaneous electrical nerve stimulation':ti,ab,kw

#4 'irritable colon'/exp OR 'irritable bowel syndrome':ti,ab,kw OR 'irritable colon syndrome':ti,ab,kw OR 'functional colonic diseases':ti,ab,kw OR 'unstable colon':ti,ab,kw

#5 #1 OR #2 OR #3

#6 #4 AND #5

Web of Science

The *Web of Science Core Collection (WoSCC)* comprises several databases. We searched the *Science Citation Index Expanded* and *Conference Proceedings Citation Index-Science* using the following search strategy to identify publications, on "topic" including title, abstract, author's keywords and KeyWords Plus®:

TS= ("Acupuncture" OR "Electroacupuncture" OR "Transcutaneous Electrical Nerve Stimulation" OR "Transcutaneous Electric Stimulation" OR "Transdermal Electrostimulation" OR "Percutaneous Electric Nerve Stimulation" OR "Percutaneous Electrical Neuromodulation") AND ("Irritable Bowel Syndrome" OR "Irritable Colon")
